# Supplementary material for: Lipocalin-2 (LCN2) Deficiency Leads to Cellular Changes in Highly Metastatic Human Prostate Cancer Cell Line PC-3
Source: Cells. 2022 Jan 13;11(2):260. doi: 10.3390/cells11020260 (PMC8773519; doi:10.3390/cells11020260)
Supplement: Supplementary file 1 [file cells-11-00260-s001.zip › cells-1524924-supplementary.pdf]

Table S1. Human primers used for RT-qPCR.

Gene	Acc. No.	Primer[1]	Source
ACTB	NM_001101.5	for: 5′-ccacgaaactaccttcaactcc-3′<br>rev: 5′-actcgtcatactcctgcttgct-3′	[1]
ATF4	NM_001675.4	for: 5′-aacaacagcaaggaggatgc-3′<br>rev: 5-tacccaacagggcatccaag-3′	Primer BLAST
CXCL8	NM_000584.4	for: 5′-aagaaaccaccggaaggaac-3′<br>rev: 5′-actccttggcaaaactgcac-3′	[2]
GAPDH	NM_002046.7	for: 5′-agccacatcgctcagacac-3′<br>rev: 5′-gcccaatacgaccaaatcc-3′	[3]
GJA1	NM_000165.5	for: 5′-acttggcgtgacttcactac-3′<br>rev: 5′-tgaaaagtactgacagccac-3′	[4]
IL1B	NM_15330.1	for: 5′-tacctgtcctgcgtgttgaa-3′<br>rev: 5′-tctttgggtaatttttgggatct-3′	ProbeLibrary
LCN2	NM_005564.5	for: 5′-ctccacctcagacctgatcc-3′<br>rev: 5′-acataccacttcccctggaat-3′	ProbeLibrary
NFKBIZ	NM_031419.4	for: 5′-ttgataccattaagtgcctaattca-3′<br>rev: 5′-ttcttcagctgccaaatgc-3′	ProbeLibrary
PCNA	NM_002592.2	for: 5′-tggagaacttggaaatggaaac-3′<br>rev: 5′-gaactggttcattcatctctatgg-3′	[5]

[1] All primers were purchased from Eurofins Genomics Germany GmbH (Ebersberg, Germany). Primer stock solutions (100 pmol/µl) were prepared in RNAse-free H_2O. Primer sequences were taken from previous publications, were designed with Universal ProbeLibrary offered by Roche Diagnostics, or were alternatively designed by use of the NCBI Primer BLAST tool.

References

1. Umebashi, K.; Tokito, A.; Yamamoto, M.; Jougasaki, M. Interleukin-33 induces interleukin-8 expression via JNK/c-Jun/AP-1 pathway in human umbilical vein endothelial cells. *PloS One* **2018**, *13*(1), e0191659. doi: 10.1371/journal.pone.0191659
2. Tancred, T.M.; Belch, A.R.; Reiman, T.; Pilarski, L.M.; Kirshner, J. (2009). Altered expression of fibronectin and collagens I and IV in multiple myeloma and monoclonal gammopathy of undetermined significance. *J. Histochem. Cytochem.* **2009**, *57*(3), 239-247. doi: 10.1369/jhc.2008.952200
3. Hokonohara, K.; Nishida, N.; Miyoshi, N.; Takahashi, H.; Haraguchi, N.; Hata, T.; Matsuda, C.; Mizushima, T.; Doki, Y.; Mori, M. (2019). Involvement of MAF1 homolog, negative regulator of RNA polymerase III in colorectal cancer progression. *Int. J. Oncol.* **2019**, *54*(3), 1001-1009. doi: 10.3892/ijo.2019.4678
4. Zhang, A.; Hitomi, M.; Bar-Shain, N.; Dalimov, Z.; Ellis, L.; Velpula, K.K.; Fraizer, G.C.; Gourdie, R.G.; Lathia, J.D. Connexin 43 expression is associated with increased malignancy in prostate cancer cell lines and functions to promote migration. *Oncotarget* **2015,** *6*(13), 11640-11651. doi: 10.18632/oncotarget.3449
5. Su, Z.; Ganbold, T.; Baigude, H. Analysis and identification of tumorigenic targets of microRNA in cancer cells by photoreactive chemical probes. *Int. J. Mol. Sci.* **2020**, *21*(4), 1545. doi: 10.3390/ijms21041545

Table S2. Primary and secondary antibodies used in Western blot analysis.

Antibody	Cat.-no	Company	Size (kDa)	Dilution	Clonality
α-tubulin (B-7)	sc-5286	Santa Cruz	55	1:1,000	m mAb
β-actin	A5441	Sigma-Aldrich	43	1:10,000	m mAb
ATF-4 (B-3)	sc-390063	Santa Cruz	40	1:500	m mAb
BIP (GRP 78) (N-20)	sc-1050	Santa Cruz	78	1:500	g pAb
CHOP/GADD153	sc-7351	Santa Cruz	30	1:500	m mAb
Connexin 43	sc-6560R	Santa Cruz	43	1:1,000	r pAb
eIF2α	#9722	Cell Signaling	38	1:1,000	r mAb
Fibronectin	AB1954	Sigma-Aldrich	262	1:3,000	r pAb
GAPDH (6C5)	sc-32233	Santa Cruz	37	1:1,000	m mAb
IL-1β (3A6)	#12242	Cell Signaling	31, (17)	1:1,000	m mAb
IL-8	sc-376750	Santa Cruz	8	1:500	m mAb
IκB-ζ	#9244	Cell Signaling	85	1:1,000	r pAb
LCN2	AF1757	R&D Systems	25, 22*	1:800	g pAb
PARP(46D11)	#9532	Cell Signaling	116, 89	1:1,000	r mAb
PCNA (6D645)	sc-71858	Santa Cruz	36	1:1,000	m mAb
p-eIF2α (Ser51)	#3597	Cell Signaling	38	1:500	r mAb
turbo GFP	TA150041	Origene	26	1:2,000	m mAb
Secondary antibodies					
goat anti-rabbit IgG (H+L), HRP	#31460	Invitrogen	-	1:5,000	r
mouse anti-goat IgG (H+L), HRP	#31400	Invitrogen	-	1:5,000	m
goat anti-mouse IgG (H+L), HRP	#31430	Invitrogen	-	1:5,000	g

Abbreviations used are: g, goat; HRP, horseradish peroxidase; m, mouse; mAb, monoclonal antibody; pAb, polyclonal antibody; r, rabbit.* 22 kDa when LCN2 is non-glycosylated via TUN, referred as *LCN2.

Table S3. Reported *LCN2* mutations in studies deposited in the TCGA database

Project	Disease type	Site	Simple somatic mutation (Affected cases)	Copy number variation (Gains)	Copy number variation (Losses)
TCGA-SKCM	Nevi and melanomas	Skin	18/469 (3.84%)	19/468 (4.06%)	7/468 (1.50%)
TCGA-UCEC	Adenomas and adenocarcinomas; cystic, mucinous and serous neoplasms; epithelial neoplasms, NOS; not reported	Corpus uteri, uterus, NOS*	14/530 (2.64%)	26/510 (5.10%)	14/510 (2.75%)
TCGA-LUSC	Squamous cell neoplasms	Bronchus and lung	7/495 (1.41%)	18/502 (3.59%)	16/502 (3.19%)
TCGA-STAD	Adenomas and adenocarcinomas; cystic, mucinous and serous neoplasm	Stomach	6/440 (1.36%)	47/432 (10.88%)	7/432 (1.62%)
TCGA-BLCA	Adenomas and adenocarcinomas; epithelial neoplasms, NOS; squamous cell neoplasms; transitional cell papillomas and carcinomas	Bladder	5/412 (1.21%)	20/408 (4.90%)	5/408 (1.23%)
TCGA-COAD	Adenomas and adenocarcinomas; complex epithelial neoplasms; cystic, mucinous and serous neoplasms; epithelial neoplasms, NOS	Colon, rectosigmoid junction	4/400 (1.00%)	4/448 (0.89%)	6/448 (1.34%)
TCGA-GBM	Gliomas; not reported	Brain	3/393 (0.76%)	13/596 (2.18%)	10/596 (1.68%)
TCGA-READ	Adenomas and adenocarcinomas; cystic, mucinous and serous neoplasms	Colon, connective, subcutaneous and other soft tissues, rectosigmoid junction, rectum, unknown	1/137 (0.73%)	4/164 (2.44%)	4/164 (2.44%)
TCGA-LAML	Myeloid leukemias	Hematopoietic and reticuloendothelial systems	1/144 (0.69%)	1/174 (0.57%)	0/174 (0.00%
CPTAC-2	Adenomas and adenocarcinomas; cystic, mucinous and serous neoplasms; ductal and lobular neoplasms; not reported; squamous cell neoplasms	Breast, colon, other and unspecified female genital organs, ovary, rectum, retroperitoneum and peritoneum	2/328 (0.61%)	0/0 (0.00%)	0/0 (0.00%)
TCGA-HNSC	Squamous cell neoplasms	Base of tongue, bones, joints and articular cartilage of other and unspecified sites, floor of mouth, gum, hypopharynx, larynx, lip, oropharynx, other and ill-defined sites in lip, oral cavity and pharynx, other and unspecified parts of mouth, other and unspecified parts of tongue, palate, tonsil	3/508 (0.59%)	22/521 (4.22%)	9/521 (1.73%)
TCGA-LIHC	Adenomas and adenocarcinomas	Liver and intrahepatic bile ducts	2/364 (0.55%)	16/371 (4.31%)	10/371 (2.70%)
TCGA-PAAD	Adenomas and adenocarcinomas; cystic,	Pancreas	1/182 (0.55%)	1/175 (0.57%)	1/175 (0.57%)

	mucinous and serous neoplasms; ductal and lobular neoplasms; epithelial neoplasms, NOS				
TCGA-ESCA	Adenomas and adenocarcinomas; cystic, mucinous and serous neoplasms; squamous cell neoplasms	Esophagus, stomach	1/184 (0.54%)	20/184 (10.87%)	2/184 (1.09%)
TCGA-BRCA	Adenomas and adenocarcinomas; adnexal and skin appendage neoplasms; basal cell neoplasms; complex epithelial neoplasms; cystic, mucinous and serous neoplasms; ductal and lobular neoplasms; epithelial neoplasms, NOS; fibroepithelial neoplasms; squamous cell neoplasms	Breast	5/986 (0.51%)	60/1.072 (5.60%)	31/1.072 (2.89%)
TCGA-SARC	Fibromatous neoplasms; lipomatous neoplasms; myomatous neoplasms; nerve sheath tumors; soft tissue tumors and sarcomas, NOS; synovial-like neoplasms	Bones, joints and articular cartilage of limbs, colon, connective, subcutaneous and other soft tissues, corpus uteri, kidney, meninges, other and unspecified male genital organs, other and unspecified parts of tongue, ovary, peripheral nerves and autonomic nervous system, retroperitoneum and peritoneum, stomach, uterus, NOS	1/237 (0.42%)	6/260 (2.31%)	24/260 (9.23%)
**TCGA-PRAD**	**Adenomas and adenocarcinomas; cystic, mucinous and serous neoplasms; ductal and lobular neoplasms**	**Prostate gland**	**2/498 (0.40%)****	**12/487 (2.46%)**	**0/487 (0.00%)**
TCGA-CESC	Adenomas and adenocarcinomas; complex epithelial neoplasms; cystic, mucinous and serous neoplasms; squamous cell neoplasms	Cervix uteri	1/289 (0.35%)	17/294 (5.78%)	1/294 (0.34%)
MMRF-COMMPASS	Plasma cell tumors	Hematopoietic and reticuloendothelial systems	2/959 (0.21%)	0/0 (0.00%)	0/0 (0.00%)
TCGA-LUAD	Acinar cell neoplasms; adenomas and adenocarcinomas; cystic, mucinous and serous neoplasms	Bronchus and lung	1/567 (0.18%)	30/513 (5.85%)	10/513 (1.95%)
CPTAC-3	Adenomas and adenocarcinomas; ductal and lobular neoplasms; gliomas; not applicable squamous cell neoplasms	Brain, bronchus and lung, kidney, other and ill-defined sites, pancreas, uterus, NOS	1/778 (0.13%)	0/0 (0.00%)	0/0 (0.00%)

Data were taken from the TCGA database (https://portal.gdc.cancer.gov/genes/ENSG00000148346?canDistTable_size=100). Please note that only the TCGA-PRAD project contains information about *LCN2* gene mutations. *NOS, not otherwise specified, ** The mutations observed in these patients were I192V or C96Y.

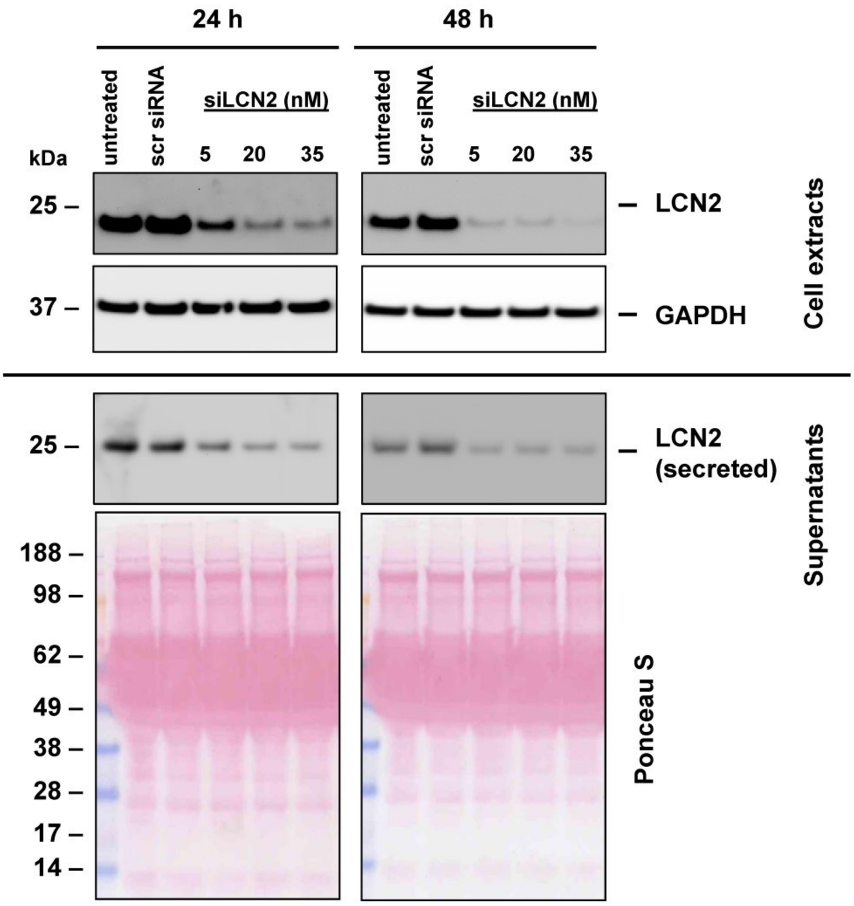

Figure S1. LCN2 levels at different times points after siRNA-mediated knockdown in PC-3 cells. LCN2 expression and secretion was analyzed 24 h and 48 h after transfection of scrambled siRNA (scr siRNA) or indicated concentrations of siLCN2. Western blot analysis showed strong reduction of LCN2 level (n≥3). GAPDH expression served as a loading control to demonstrate equal protein. Secreted LCN2 levels are shown with corresponding Ponceau S stain.

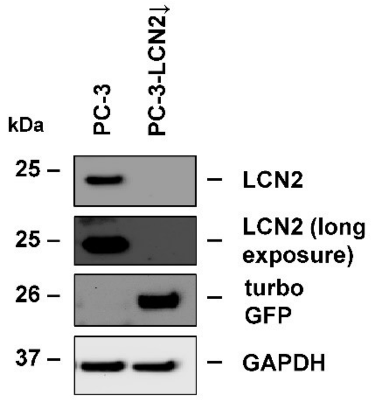

Figure S2. Depletion of LCN2 *via* CRISPR/Cas9 in PC-3 cells. PC-3 cells and stable a pool of LCN2-deficient PC-3 cells were grown in basal conditions for subsequent protein analysis. Representative Western blot (n≥3) show LCN2 expression in PC-3 cells but not in LCN2-deficient PC-3 cells (PC-3-LCN2↓). Turbo GFP expression was detectable in LCN2-deficient cells. GAPDH expression served as a control for equal protein loading.

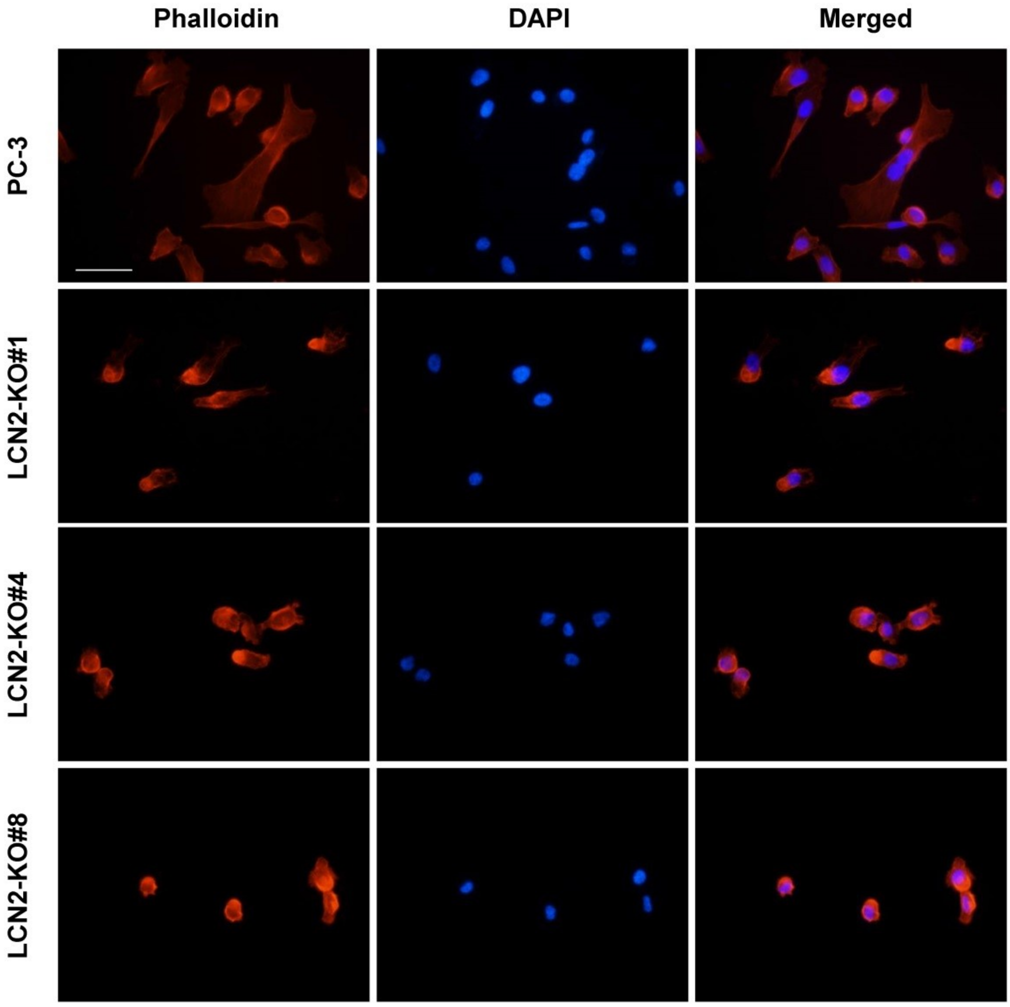

Figure S3. Cytoskeleton organization in LCN2-deficent cell lines. F-actin stress fibers were stained with Phalloidin-Rhodamine (*red*) in PC-3 and three different LCN2-KO cell lines (n=2). Nuclei were counterstained with DAPI (*blue*). Magnification: 400x, scale bar: 50 µm.

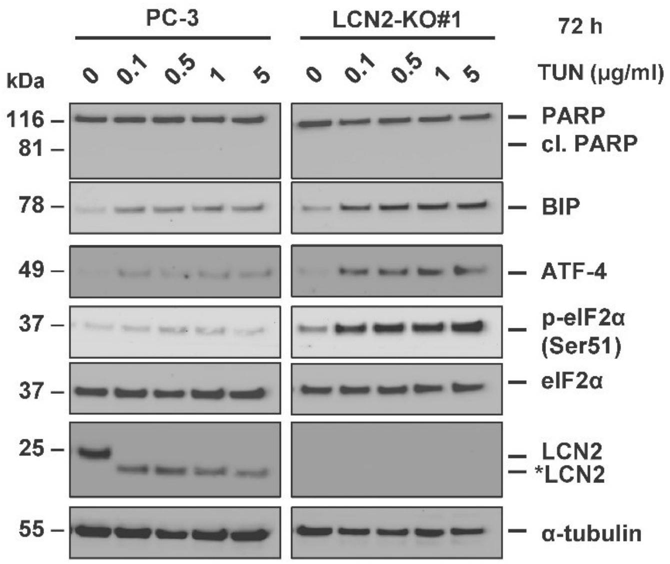

Figure S4. Prolonged tunicamycin (TUN)-induced ER stress in PCa cells. PC-3 and LCN2-KO#1 cells were stimulated with indicated concentrations of TUN for 72 h and harvested for protein analysis. Representative Western blot (n=2) showed stronger activation of different UPR markers in LCN2-KO#1 than PC-3 cells. α-tubulin served as a control for equal protein loading.

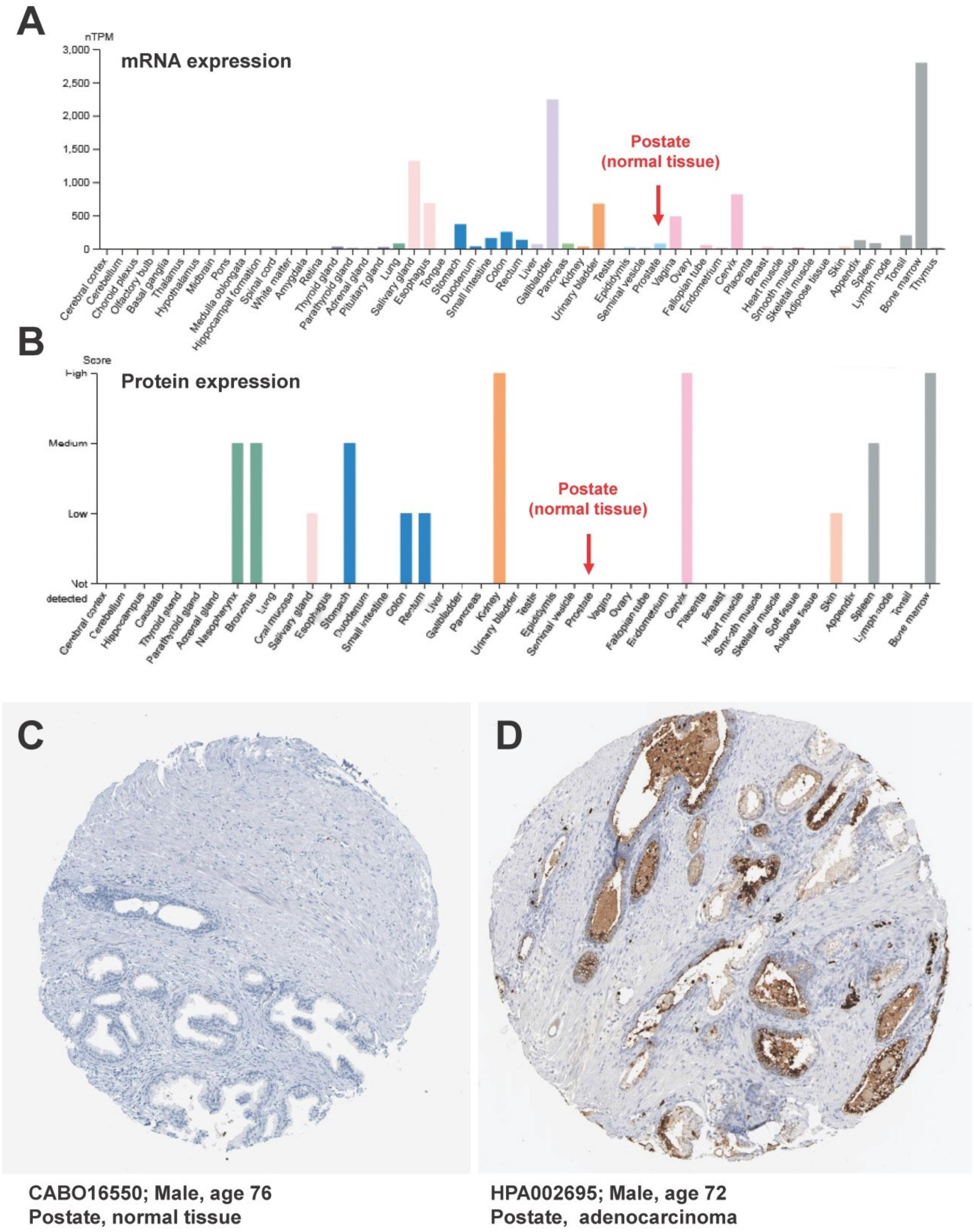

Figure S5. Expression of LCN2 in normal and cancerous human prostate tissue. Normal human prostate tissue expresses only low or no quantities of LCN2 mRNA (**A**) and protein (**B**). (**C,D**) In comparison to normal prostate tissue, tissue obtained from a patient suffering from an adenocarcinoma shows extremely high expression of LCN2 in immunohistochemical analysis. All data presented in this figure were taken from the Human Protein Atlas (https://www.proteinatlas.org/).
